# Correlation of Waist Circumference–Mediated Glycosylated Hemoglobin Levels With New‐Onset BADL Disability

**DOI:** 10.1155/jdr/6628845

**Published:** 2026-08-26

**Authors:** Jing Ren, Tian Lv, Qinna Mao, Yong Zhang

**Affiliations:** ^1^ School of Medicine, Shaoxing University, Shaoxing, China, usx.edu.cn; ^2^ Department of Neurology, Zhuji Affiliated Hospital of Wenzhou Medical University, Zhuji, China; ^3^ Department of Nursing, Zhuji Affiliated Hospital of Wenzhou Medical University, Zhuji, China

**Keywords:** glycated hemoglobin, mediation effect, new-onset BADL disability, waist circumference

## Abstract

**Background:**

Impairment in basic activities of daily living (BADLs) is strongly associated with chronic disease. Glycosylated hemoglobin (HbA1c) reflects long‐term glycemic control and is related to several chronic diseases and central obesity. However, the relationship between HbA1c and BADL disability remains unclear. This study examined the association between HbA1c levels and new‐onset BADL disability and explored the mediating role of waist circumference.

**Methods:**

A longitudinal research design was used to obtain data from the China Health and Retirement Longitudinal Study (CHARLS). This study included 4318 middle‐aged and older adults without BADL disability at baseline (2011), and they were followed up until 2020. Participants were categorized into quartiles based on baseline HbA1c levels (Q1–Q4). The Kaplan–Meier curves, Cox regression, and restricted cubic splines were used to assess the association between HbA1c levels and new‐onset BADL disability. Mediation analysis was employed to evaluate the role of waist circumference.

**Results:**

During an average follow‐up period of 8.19 years, the hazard ratios (HRs) for BADL disability were higher in the Q1, Q2, and Q4 groups than in the Q3 group (*p* < 0.05). Each 1‐unit increase in HbA1c was associated with an 11% higher risk of BADL disability (HR: 1.11; 95% CI: 1.02–1.20). A nonlinear threshold effect was observed at HbA1c levels of 5.2%, with a significant increase in the risk of BADL disability beyond this threshold (HR: 1.23; 95% CI: 1.13–1.33). Mediation analysis showed that waist circumference accounted for 20% of the overall effect of HbA1c levels on the risk of BADL disability.

**Conclusion:**

Elevated HbA1c levels (particularly above 5.2%) predicted an increased risk of developing BADL disability, and waist circumference partially mediated the effect of HbA1c levels on BADL disability. These suggest that glycemic control with waist circumference management may be an important strategy for reducing the risk of BADL disability.

## 1. Introduction

The demographic transition to an aging population has become an urgent global issue, and China is one of the countries in the world facing this problem most severely and rapidly [1]. In this context, the functional health of middle‐aged and older people has been identified as an important public health issue. Basic activities of daily living (BADLs) have been identified as an important indicator of functional health in older adults. These activities, which include dressing, eating, and bathing, are closely associated with an individual′s level of independence and quality of life [2]. BADL disability not only significantly reduces the quality of life of middle‐aged and older adults [3] but is also strongly associated with adverse health outcomes, including depression [4], hospitalization [5], and mortality [6, 7]. As of 2021, approximately 16% of the global population was affected by BADL disability, with 34.4% of individuals aged 60 years and over experiencing this condition [8].

The risk factors contributing to BADL disability are complex and multifaceted, with chronic diseases playing a particularly important role [9]. Of all the chronic diseases out there, diabetes is one of the most complicated. It affects several different systems in the body. Prolonged poor glycemic control has been shown to lead to multiple chronic complications, including retinopathy, nephropathy, and cardiovascular disease. It has also been reported to potentially impair physical function and the ability to perform activities of daily living through mechanisms such as accelerated aging, inflammation, and oxidative stress [10, 11]. Glycated hemoglobin (HbA1c) is an important biomarker for assessing and diagnosing diabetes, reflecting average blood glucose levels over the previous 2–3 months [12]. Studies have suggested that elevated HbA1c levels in diabetic patients may be associated with an increased risk of BADL disability [13]. However, there is limited evidence of this association in nondiabetic individuals. Indeed, clinical and epidemiological studies have suggested that variations in HbA1c levels, even within the normal range (5.7%–6.5%), may still pose potential health risks [14, 15]. Therefore, further research is needed to clarify the link between HbA1c levels and the risk of developing BADL disability.

Furthermore, waist circumference is recognized as a key predictor of metabolic syndrome and an essential variable in studies investigating health outcomes among middle‐aged and older adults [16]. An increased waist circumference has been shown to have a strong correlation with insulin resistance and chronic inflammation. Moreover, it has been suggested that an increase in waist circumference may accelerate functional decline by disrupting energy metabolism [17]. Existing literature suggests that waist circumference may act as an intermediate variable between HbA1c levels and functional health [18]. However, this hypothesis has not yet been systematically investigated. Previous research has also examined the relationship between waist circumference and blood glucose levels. For example, Wu et al. [19] found a significant positive linear correlation between changes in waist circumference and fasting glucose levels in individuals at high risk of cardiovascular disease. This finding highlights the potential role of waist circumference in mediating the association between metabolic indicators and health outcomes. However, the precise role of waist circumference in the relationship between HbA1c levels and functional health remains unclear. Additionally, elevated HbA1c levels have been linked to abnormal fat distribution, particularly the accumulation of visceral fat, which leads to an increase in waist circumference [20]. Individuals with a larger waist circumference are more likely to have reduced muscle mass and increased chronic inflammation, which may impair physical mobility [21, 22].

Based on these findings, we hypothesized that HbA1c levels may further exacerbate BADL disability by increasing waist circumference, which may partially mediate the association between HbA1c levels and BADL disability. This study systematically investigated the relationship between HbA1c levels and the risk of BADL disability in a middle‐aged and elderly Chinese population, with a particular focus on the potential mediating role of waist circumference.

## 2. Methods

### 2.1. Data Sources and Study Population

The present study employed a cohort research design, utilizing follow‐up data from the China Health and Retirement Longitudinal Study (CHARLS). The CHARLS is a large‐scale, long‐term survey project hosted by the National Development Research Institute of Peking University and implemented by Peking University′s China Center for Social Science Surveys. The CHARLS collected high‐quality microdata on middle‐aged and older adults (aged 45 and over) and their households across 28 provinces in China [23]. The CHARLS study was approved by the Biomedical Ethics Review Board of Peking University (approval no. IRB00001052‐11015), and all participants provided written informed consent before participation. Relevant study data are available at http://charls.pku.edu.cn/.

### 2.2. Research Design

The present study examined the association between HbA1c levels in individuals without BADL disability in 2011 and the subsequent onset of BADL disability during follow‐ups in 2015, 2018, and 2020. Furthermore, the study analyzed the moderating effects of individual demographic factors, health behaviors, laboratory indicators, and other chronic disease conditions on this relationship.

**Figure 1:**
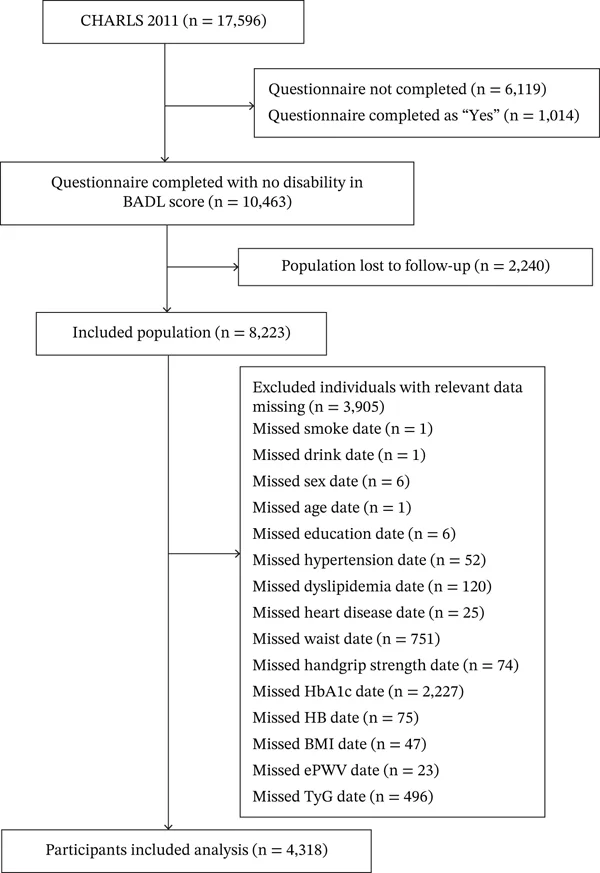
Flow diagram for participants included in the study.

The inclusion criteria for this study were determined based on its purpose and content. Participants were selected from the 2011 CHARLS baseline survey and had to be aged 45 years or older. They could not have reported any limitations on any items in the BADL questionnaire at baseline. Participants were required to have participated in at least one follow‐up survey (in 2015, 2018, or 2020) and to have complete data on the study variables. A total of 17,596 participants were initially enrolled in CHARLS Wave 1, but some were excluded due to meeting one or more of the following criteria: not completing the baseline questionnaire (*n* = 6119), having BADL disability at baseline (*n* = 1014), being lost to follow‐up (*n* = 2240), or having missing key variables (*n* = 3905). Ultimately, 4318 eligible participants were included in the analysis (Figure 1).

### 2.3. Outcome Variable

The outcome of this study was the onset of the first BADL disability, with functional status being assessed using the BADL questionnaire [24, 25]. The CHARLS survey asked participants whether they needed assistance with six BADLs: dressing, bathing, eating, getting in and out of bed, using the toilet, and controlling urination and defecation [26]. For each BADL item, respondents could choose from the following options: (1) no difficulty at all, (2) difficult but still able to do it, (3) difficult and need help, and (4) unable to do it. These responses were scored from 0 to 3 [27, 28]. Based on previous studies, BADL status was classified as having no limitations or having at least one limitation [29, 30]. In this study, all participants had no BADL limitations at baseline. Those who developed one or more limitations during the follow‐up period were classified as having “new‐onset BADL disability.” For individuals who developed a BADL disability, the time of onset was determined by the interval between the earliest reported year of onset and the baseline year.

### 2.4. Exposing Variable

HbA1c is a key indicator for assessing an individual′s average glycemic control over the past 2–3 months, with higher HbA1c levels typically reflecting poorer glycemic control. According to the World Health Organization (WHO) criteria, an HbA1c level of ≥ 6.5% is one of the diagnostic criteria for diabetes mellitus [31]. In this study, blood samples for biochemical analysis were collected by medically trained staff at the Chinese Centre for Disease Control and Prevention (CDC). Participants were instructed to fast overnight before sample collection. Blood samples were immediately stored at 4°C and transported to the CDC for testing within 2 weeks, where HbA1c levels were analyzed using borate affinity liquid chromatography [32].

### 2.5. Covariates

All covariates were collected at baseline and included demographics, health behaviors, laboratory tests, and other chronic diseases. Demographic variables included age (< 60 years or ≥ 60 years), sex, education level (secondary school or higher), and residence (rural or urban). Height, waist circumference, and weight were measured after participants removed their shoes and heavy clothing, with height and waist circumference recorded to the nearest 0.1 cm and weight to the nearest 0.1 kg. Health behaviors included drinking status (never, less than once a month, or more than once a month) and smoking status (never smoked, former smoker but quit, or current smoker). Body mass index (BMI) was calculated using the formula weight/height^2^ (kg/m^2^) and categorized according to Asian population standards into three groups: normal weight (BMI < 24.0 kg/m^2^), overweight (BMI ≥ 24.0 kg/m^2^), and obese (BMI ≥ 28.0 kg/m^2^) [33]. Grip strength was measured using a special handheld dynamometer, with each hand tested twice, and the maximum value from both measurements was recorded as the final result. Laboratory tests included hemoglobin, glucose, creatinine, uric acid, total cholesterol (TC), high‐density lipoprotein (HDL), low‐density lipoprotein (LDL), triglyceride (TG), and HbA1c. Venous blood samples were collected by medically trained staff using standard phlebotomy procedures, and biochemical tests were performed. Participants were instructed to fast for at least 8 h before blood sampling.

The diagnosis of hypertension was based on self‐reported history of hypertension, use of any antihypertensive medication, and/or mean systolic/diastolic blood pressure (SBP/DBP) ≥ 140/90 mmHg [34]. Diabetes was defined as fasting plasma glucose (FPG) ≥ 126 mg/dL, HbA1c ≥ 6.5%, a self‐reported history of diabetes diagnosis, and/or use of antihyperglycemic medication. Dyslipidemia was diagnosed based on a self‐reported history of dyslipidemia, current use of lipid‐lowering medication, and/or the presence of any of the following: TC ≥ 240 mg/dL, TG ≥ 150 mg/dL, HDL < 40 mg/dL, or LDL ≥ 160 mg/dL [35]. Heart disease, including coronary heart disease, myocardial infarction, and angina pectoris, was determined by physician diagnosis or medical history.

### 2.6. Statistical Analysis

Normally distributed continuous data were presented as mean ± standard deviation and analyzed for statistical significance using a *t*‐test. Categorical variables were expressed as frequencies and percentages and compared using a chi‐squared test.

Participants were divided into four groups based on their HbA1c quartile levels: first quartile group (Q1): 3.7*%* < HbA1c < 4.9*%*; second quartile group (Q2): 4.9*%* < HbA1c < 5.2*%*; third quartile group (Q3): 5.2*%* < HbA1c < 5.4*%*; and fourth quartile group (Q4): 5.4*%* < HbA1c < 12.8*%*. HbA1c levels (quartiles and continuous variables) were analyzed as independent variables. Based on HbA1c grouping, the Kaplan–Meier method was used to estimate the cumulative incidence of BADL disability, and differences were compared using the log‐rank test. Cox proportional hazard regression analyses were conducted to investigate the relationship between baseline HbA1c levels and the occurrence of new‐onset BADL disability and to calculate the hazard ratio (HR) and 95% confidence interval (CI). Three models were estimated. The crude model was unadjusted. Model 1 was adjusted for age and sex. Model 2 was further adjusted for education level, drinking status, and smoking status based on Model 1, and Model 3 was additionally adjusted for hypertension, dyslipidemia, LDL, diabetes, heart disease, grip strength, height, and weight based on Model 2. Given that grip strength is an internationally recognized indicator that objectively reflects an individual′s overall muscle function and physical health status, this study incorporated grip strength as a covariate into the model to control for its potential confounding effects between HbA1c and new‐onset BADL disability. As Q3 had the highest concentration of data points, it was used as the reference group. A multivariable‐adjusted Cox regression model with restricted cubic splines (RCSs) was used to explore the nonlinear relationship between baseline HbA1c levels and the risk of new‐onset BADL disability. In the nonlinear model, the inflection point was determined using maximum likelihood estimation. A two‐stage regression model was also used to assess the nonlinear association between HbA1c levels and the risk of new‐onset BADL disability before and after the inflection point. Subgroup analyses were stratified by baseline age, sex, education, smoking status, drinking status, dyslipidemia, diabetes mellitus, heart disease, and BMI to further assess the association between HbA1c levels and new‐onset BADL disability in different populations. In addition, a general mediation model was constructed to examine the role of waist circumference as a mediator in the relationship between HbA1c levels and new‐onset BADL disability. Mediation analyses were performed using the bootstrap method (5000 iterations) proposed by Preacher et al. [36].

To appropriately handle missing values for covariates in the study, we used the “mice” package in R to impute the missing values using multiple chain interpolation. All statistical analyses were performed using R (Version 4.4.1). A two‐sided *p* value < 0.05 was considered to indicate statistical significance.

## 3. Results

### 3.1. General Characteristics of Participants

The baseline characteristics of participants grouped by HbA1c quartiles are summarized in Table 1. The mean age of the 4318 participants was 59.43 ± 9.26 years, and 2731 (63.25%) were female. The onset of BADL impairment occurred at 8.19 ± 2.00 years of follow‐up. The Q4 group experienced the earliest onset of BADL impairment (8.04 ± 2.15), whereas the Q3 group experienced the latest (8.43 ± 1.73). Significant differences in age, weight, BMI, and waist circumference were observed between participants in the lowest and highest HbA1c quartiles (all *p* < 0.05), with those in the higher quartiles being older, having a higher BMI, and a significantly larger waist circumference. In addition, the prevalence of diabetes, hypertension, dyslipidemia, and heart disease was higher in the higher HbA1c quartiles (all *p* < 0.05). Higher levels of hemoglobin, glucose, uric acid, total TC, LDL, and TG were also observed in the higher HbA1c quartiles (all *p* < 0.05). However, no significant differences in educational level, smoking, or alcohol consumption were observed across the different HbA1c quartiles.

**Table 1:** The characteristics of all participants classified by HbA1c status.

Variable	Total (*n* = 4318)	Q3 (*n* = 400)	Q1 (*n* = 876)	Q2 (*n* = 1660)	Q4 (*n* = 1382)	Statistic	*p*value
Age (years)	59.43 ± 9.26	59.46 ± 8.55	58.61 ± 10.06	59.15 ± 9.18	60.28 ± 8.94	6.70	< 0.001
Hemoglobin (g/dL)	14.30 ± 2.28	14.46 ± 2.09	14.01 ± 2.30	14.29 ± 2.49	14.45 ± 2.02	7.68	< 0.001
Glucose (mg/dL)	109.06 ± 32.48	105.49 ± 18.08	99.70 ± 15.90	102.31 ± 17.15	124.14 ± 48.46	166.12	< 0.001
Creatinine (mg/dL)	0.76 ± 0.19	0.75 ± 0.18	0.76 ± 0.22	0.76 ± 0.17	0.76 ± 0.20	0.35	0.79
Uric acid (mg/dL)	4.31 ± 1.20	4.44 ± 1.25	4.23 ± 1.23	4.24 ± 1.13	4.41 ± 1.24	8.44	< 0.001
TC (mg/dL)	195.52 ± 39.01	195.92 ± 38.01	187.05 ± 37.78	194.78 ± 38.32	201.66 ± 39.84	25.82	< 0.001
HDL (mg/dL)	51.23 ± 14.96	52.52 ± 16.31	51.34 ± 14.41	51.42 ± 14.50	50.58 ± 15.41	1.97	0.12
LDL (mg/dL)	118.34 ± 34.66	117.41 ± 34.61	113.08 ± 32.99	118.12 ± 34.56	122.22 ± 35.37	12.69	< 0.001
TG (mg/dL)	132.67 ± 110.70	126.15 ± 83.87	119.02 ± 102.38	129.05 ± 99.97	147.56 ± 131.37	13.95	< 0.001
Height (m)	1.57 ± 0.08	1.56 ± 0.09	1.57 ± 0.08	1.57 ± 0.08	1.56 ± 0.08	2.48	0.06
Weight (kg)	58.45 ± 11.70	58.85 ± 12.98	57.15 ± 10.68	57.75 ± 11.21	60.01 ± 12.34	14.00	< 0.001
BMI (kg/m^2^)	23.73 ± 4.04	23.98 ± 4.80	23.06 ± 3.52	23.41 ± 3.87	24.48 ± 4.18	28.33	< 0.001
Waist (cm)	84.76 ± 12.83	85.18 ± 12.44	82.20 ± 13.32	84.07 ± 12.37	87.09 ± 12.78	29.11	< 0.001
Time (years)	8.19 ± 2.00	8.43 ± 1.73	8.27 ± 1.88	8.21 ± 1.98	8.04 ± 2.15	4.89	< 0.01
HbA1c (%)	5.29 ± 0.78	5.30 ± 0.00	4.65 ± 0.17	5.05 ± 0.11	5.98 ± 1.03	1065.57	< 0.001
Handgrip strength (kg)	29.50 ± 9.60	29.50 ± 11.24	29.85 ± 9.71	29.61 ± 9.48	29.14 ± 9.14	1.12	0.34
Sex, *n* (%)						1.73	0.63
Female	2731 (63.25)	261 (65.25)	540 (61.64)	1055 (63.55)	875 (63.31)		
Male	1587 (36.75)	139 (34.75)	336 (38.36)	605 (36.45)	507 (36.69)		
Education, *n* (%)						3.49	0.32
> High school	3308 (76.61)	316 (79.00)	659 (75.23)	1260 (75.90)	1073 (77.64)		
Middle school	1010 (23.39)	84 (21.00)	217 (24.77)	400 (24.10)	309 (22.36)		
Residence, *n* (%)						6.70	0.08
Rural	2988 (69.20)	266 (66.50)	607 (69.29)	1183 (71.27)	932 (67.44)		
Urban	1330 (30.80)	134 (33.50)	269 (30.71)	477 (28.73)	450 (32.56)		
Smoke, *n* (%)						9.31	0.16
Former, now quit	331 (7.67)	21 (5.25)	70 (7.99)	131 (7.89)	109 (7.89)		
Never smoke	2909 (67.37)	270 (67.50)	565 (64.50)	1127 (67.89)	947 (68.52)		
Still smoke	1078 (24.97)	109 (27.25)	241 (27.51)	402 (24.22)	326 (23.59)		
Drink, *n* (%)						11.95	0.06
< 1 m	311 (7.20)	31 (7.75)	74 (8.45)	121 (7.29)	85 (6.15)		
> 1 m	884 (20.47)	79 (19.75)	199 (22.72)	348 (20.96)	258 (18.67)		
No	3123 (72.33)	290 (72.50)	603 (68.84)	1191 (71.75)	1039 (75.18)		
Dyslipidemia, *n* (%)						18.06	< 0.001
No	3864 (89.49)	366 (91.50)	796 (90.87)	1505 (90.66)	1197 (86.61)		
Yes	454 (10.51)	34 (8.50)	80 (9.13)	155 (9.34)	185 (13.39)		
Diabetes, *n* (%)						398.30	< 0.001
No	3646 (84.44)	351 (87.75)	817 (93.26)	1531 (92.23)	947 (68.52)		
Yes	672 (15.56)	49 (12.25)	59 (6.74)	129 (7.77)	435 (31.48)		
Heart disease, *n* (%)						12.30	< 0.01
No	3757 (87.01)	351 (87.75)	790 (90.18)	1439 (86.69)	1177 (85.17)		
Yes	561 (12.99)	49 (12.25)	86 (9.82)	221 (13.31)	205 (14.83)		
Hypertension, *n* (%)						22.56	< 0.001
No	3109 (72.00)	286 (71.50)	665 (75.91)	1224 (73.73)	934 (67.58)		
Yes	1209 (28.00)	114 (28.50)	211 (24.09)	436 (26.27)	448 (32.42)		

*Note:* Data presented as mean ± standard deviation for continuous variables and as number and proportion for categorical variables. Drinking status: < 1 m, less than 1 time per month; > 1 m, more than 1 time per month; no, no drinking.

Abbreviations: BMI, body mass index; HbA1c, glycated hemoglobin; HDL, high‐density lipoprotein; LDL, low‐density lipoprotein; TC, total cholesterol; TG, triglyceride.

### 3.2. Correlation Between Baseline HbA1c Levels and New‐Onset BADL Disability

**Figure 2:**
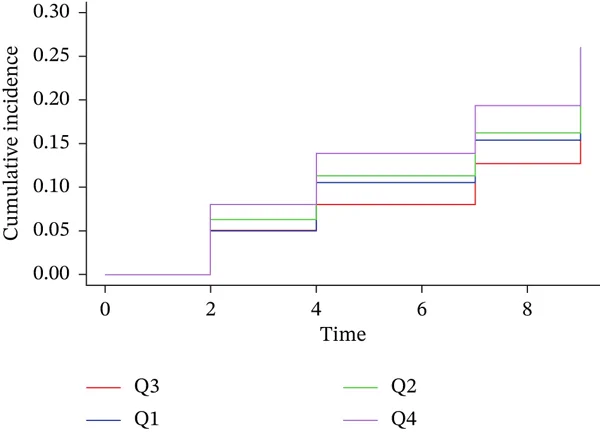
The Kaplan–Meier analysis for new‐onset BADL disability was based on HbA1c quartiles. The vertical axis indicates cumulative incidence (%), and the horizontal axis is time (years).

The Kaplan–Meier cumulative incidence curve analysis showed a gradual increase in new‐onset BADL disability events over time in the Q1, Q2, Q3, and Q4 groups, with significant differences between groups (Figure 2, log‐rank test, *p* = 0.001). The cumulative incidence increased more significantly in the Q1, Q2, and Q4 groups than in the Q3 group.

A significant association between baseline HbA1c levels and new‐onset BADL disability was confirmed using Cox proportional hazards models, with baseline HbA1c levels analyzed both as a continuous variable and as a categorical variable (quartiles). When analyzed as a continuous variable, each 1‐unit increase in the baseline HbA1c level was associated with an 11% higher risk of new‐onset BADL disability (HR: 1.11; 95% CI 1.02–1.20) in Model 3, after adjusting for potential confounders (Table 2).

**Table 2:** The relationship between HbA1c levels and new‐onset BADL disability.

Variable	Crude model HR (95% CI)	*p*	Model 1 HR (95% CI)	*p*	Model 2 HR (95% CI)	*p*	Model 3 HR (95% CI)	*p*
HbA1c	1.19 (1.12, 1.27)	< 0.001	1.16 (1.09, 1.23)	< 0.001	1.15 (1.08, 1.23)	< 0.001	1.11 (1.02, 1.20)	0.01
HbA1c Q								
Q3	Ref		Ref		Ref		Ref	
Q1	1.32 (1.00, 1.74)	0.05	1.35 (1.03, 1.79)	0.03	1.38 (1.04, 1.82)	0.02	1.47 (1.11, 1.94)	0.01
Q2	1.36 (1.05, 1.76)	0.02	1.39 (1.08, 1.80)	0.01	1.41 (1.09, 1.82)	0.01	1.49 (1.15, 1.93)	0.003
Q4	1.62 (1.25, 2.09)	< 0.001	1.54 (1.19, 1.99)	0.001	1.54 (1.19, 2.00)	0.001	1.50 (1.15, 1.95)	0.002
*p* for trend		< 0.001		0.002		0.003		0.02

*Note:* Data are presented as hazard ratio (HR) with 95% confidence interval (CI) and *p* value. Models: crude model, unadjusted; Model 1, adjusted for age and sex; Model 2, adjusted for age, sex, education, drink status, and smoke status; Model 3, adjusted for age, sex, education, drink status, smoke status, hypertension, dyslipidemia, LDL, diabetes, heart disease, handgrip strength, weight, and height.

When HbA1c was analyzed as a categorical variable with the Q3 group as the reference group, in the fully adjusted model (Model 3), compared with the Q3 group, the Q1 group had a 47% increased risk of new‐onset BADL disability (HR: 1.47; 95% CI: 1.11–1.94; *p* = 0.01), the risk of new‐onset BADL disability in the Q2 group was 49% higher (HR: 1.49; 95% CI: 1.15–1.93; *p* = 0.003), and the risk in the Q4 group was 50% higher (HR: 1.50; 95% CI: 1.15–1.95; *p* = 0.002). Trend analysis revealed a significant positive trend between HbA1c quartile levels and the risk of new‐onset BADL disability (*p* = 0.02) (Table 2).

**Figure 3:**
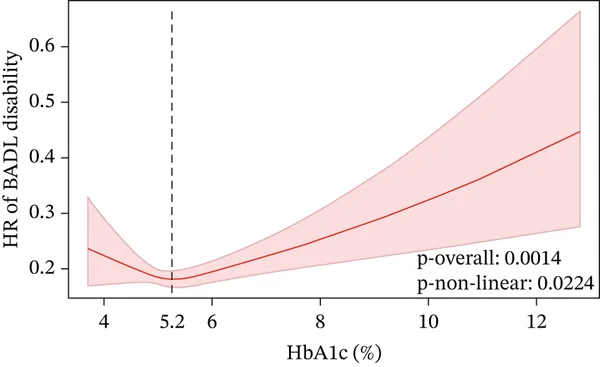
The restricted cubic spline analysis of the relationship between HbA1c levels and new‐onset BADL disability.

To further explore the potential nonlinear relationship between HbA1c levels and the risk of new‐onset BADL disability, an RCS regression model was employed (Figure 3). The results showed a significant nonlinear association between HbA1c levels and the risk of new‐onset disability in BADL (*p* − overall = 0.0014, *p* − nonlinear = 0.0224). The nonlinear relationship was further analyzed using a two‐stage linear regression model (Table 3), and the results showed that the inflection point for HbA1c was 5.2%. When HbA1c < 5.2*%*, no significant association was observed between HbA1c levels and the risk of new‐onset BADL disability (HR: 1.24; 95% CI: 0.82–1.88; *p* = 0.311); however, when HbA1c was ≥ 5.2%, each 1‐unit increase in HbA1c was associated with a significant 23% increase in the risk of new‐onset BADL disability (HR: 1.23; 95% CI: 1.13–1.33; *p* < 0.001).

**Table 3:** The two‐stage linear regression model of the relationship between HbA1c levels and new‐onset BADL disability.

Interval	HR (95% CI)	*p*value
Fitting by two‐piecewise model		
Inflection points of HbA1c	5.2%	
< 5.2%	1.24 (0.82, 1.88)	0.311
≥ 5.2%	1.23 (1.13, 1.33)	< 0.001
Log‐likelihood ratio *p* value		< 0.001

### 3.3. Subgroup Analysis

The association between HbA1c levels and new‐onset BADL disability was further examined in several subgroups (Table 4), with age (*p* = 0.021) and drinking status (*p* = 0.035) identified as significant moderators of this relationship. In contrast, no significant interactions were observed in the other subgroups.

**Table 4:** The subgroup analysis of the relationship between HbA1c levels and new‐onset BADL disability.

Character	HR (95% CI)	*p*	*p*for interaction
Age (years)			0.021
< 60	1.29 (1.17, 1.43)	< 0.001	
≥ 60	1.10 (1.01, 1.20)	0.024	
Sex			0.764
Female	1.20 (1.11, 1.30)	< 0.001	
Male	1.17 (1.05, 1.31)	0.005	
Education			0.089
Middle school	1.34 (1.16, 1.54)	< 0.001	
> High school	1.16 (1.08, 1.25)	< 0.001	
Smoke			0.778
Never smoke	1.21 (1.12, 1.30)	< 0.001	
Still smoke	1.17 (1.03, 1.32)	0.019	
Former, now quit	1.11 (0.83, 1.49)	0.483	
Drink			0.035
No	1.23 (1.15, 1.32)	< 0.001	
< 1 m	1.16 (0.90, 1.50)	0.267	
> 1 m	0.92 (0.73, 1.17)	0.510	
Dyslipidemia			0.767
No	1.18 (1.10, 1.27)	< 0.001	
Yes	1.21 (1.04, 1.41)	0.015	
Diabetes			0.629
No	1.10 (0.91, 1.32)	0.314	
Yes	1.15 (1.06, 1.25)	< 0.001	
Heart disease			0.346
No	1.20 (1.12, 1.29)	< 0.001	
Yes	1.11 (0.95, 1.30)	0.191	
BMI			0.647
Obesity	1.22 (1.06, 1.41)	0.007	
Nonobesity	1.18 (1.10, 1.27)	< 0.001	

### 3.4. Mediation Analysis

**Figure 4:**
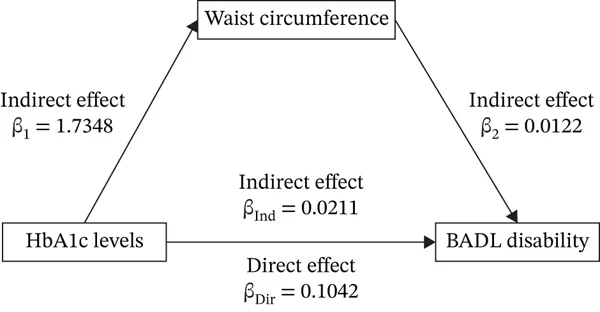
Mediation analysis of waist circumference in the correlation between HbA1c levels and new‐onset BADL disability.

Mediation analyses indicated that waist circumference partially mediated the association between HbA1c levels and new‐onset BADL disability (Figure 4). HbA1c levels had a significant effect on waist circumference (*β*_1_ = 1.73, *p* < 0.001), while waist circumference significantly influenced the incidence of new‐onset BADL disability (regression coefficient *β*_2_ = 0.0122, *p* < 0.001). The indirect effect (*β*_Ind_) of HbA1c levels on new‐onset BADL disability was 0.0211 (*p* < 0.001), while the direct effect (*β*_Dir_) was 0.1042 (*p* < 0.001). Mediation effect analysis further showed that waist circumference accounted for 20% (95% CI: 9.1%–31.4%) of the total effect of HbA1c levels on new‐onset BADL disability.

## 4. Discussion

This study is the first to systematically investigate the longitudinal association between HbA1c levels and new‐onset BADL disability, leveraging data from the CHARLS. Through mediation analysis, it further revealed the critical role of waist circumference in mediating this relationship. Meanwhile, China′s population is aging rapidly, meaning that middle‐aged individuals represent a high‐risk reserve population for functional impairment in older age. To identify early risk factors for functional decline and provide opportunities for preventive interventions earlier, the analysis included individuals aged 45 and above from the CHARLS database.

The results of this study showed that elevated HbA1c levels were significantly associated with an increased risk of new‐onset BADL disability. This observation is consistent with previous evidence, such as the study by Shao et al. [37], which found a robust association between long‐term HbA1c variability and functional decline. This relationship may be mediated by metabolic disturbances, including chronic neuropathy, reduced muscle mass, and cardiovascular disease, which result from prolonged poor glycemic control and can significantly impair an individual′s ability to perform activities of daily living [38–40]. In further support, Mutambudzi et al. [41] reported that baseline HbA1c levels significantly predicted 8‐year trajectories of functional decline in adults aged ≥ 50 years. Specifically, participants with HbA1c levels ≥ 6.5% had a 63% greater likelihood (HR: 1.63; 95% CI: 1.25–2.11) of progressing along a “high‐increasing trajectory” of accelerated dysfunction compared to those with lower levels. Notably, although elevated HbA1c may have a biologically plausible influence on functional disability in nondiabetic populations, this effect lacks statistical significance in the cohorts [42]. This discrepancy may be due to the subclinical progression of metabolic dysregulation that has not yet manifested as overt functional impairment. Furthermore, in our sample, individuals in the low HbA1c group tended to have lower body weight, poorer nutritional status, and a relatively higher prevalence of chronic diseases. Therefore, low HbA1c indicates underlying poor health rather than “good glycemic control.” Consequently, proactive glycemic management in prediabetic and high‐risk individuals is emerging as a critical preventive strategy against the onset of functional disability. A 12‐year longitudinal study from Sweden confirmed that, in addition to diabetes, prediabetes was also associated with accelerated functional decline and disability [43]. In this study, we found that the lowest risk of basic functional impairment was associated with HbA1c levels in the range of approximately 5.2%–5.4% (Q3). Both lower and higher values were found to be associated with an increased risk. This pattern is physiologically plausible in middle‐aged and older adults: Excessively low HbA1c levels may indicate malnutrition or chronic disease [44], while excessively high levels suggest poor glycemic control and metabolic dysfunction, both of which could contribute to functional decline. Simultaneously, our study found a nonlinear relationship between HbA1c levels and new‐onset BADL disability with an inflection point at 5.2%, and elevated HbA1c levels significantly increased the risk of new‐onset BADL disability when HbA1c levels were greater than 5.2% (*p* < 0.0001). This finding has important clinical value in moving preventive measures from late‐stage disease management to early‐stage screening and intervention. Reducing HbA1c levels through early dietary and lifestyle changes and maintaining them at around 5.2% significantly reduces the risk of new‐onset BADL disability.

Subgroup analyses showed that the effect of elevated HbA1c levels on new‐onset BADL disability was more pronounced in the younger group (< 60 years old). In contrast, this effect was relatively attenuated in the older group (≥ 60 years old), consistent with the findings of Hsu et al. [45] and Yau et al. [46]. This reflects the greater sensitivity of younger individuals to changes in HbA1c levels due to their greater compensatory capacity. The long‐term impact of elevated HbA1c levels may exert a more substantial cumulative detrimental effect on the health of younger individuals. Conversely, in older adults, additional risk factors—such as multiple comorbidities or chronic inflammatory states—may further obscure or attenuate the direct impact of HbA1c levels on functional decline. These findings highlight the importance of early lifestyle interventions for middle‐aged individuals with elevated HbA1c levels, which can help reduce the risk of future functional decline. In addition, the association between HbA1c levels and new‐onset BADL disability was more significant in the never‐drinkers group. This suggests that alcohol consumption may modulate the direct effects of elevated HbA1c levels on functional decline in part by affecting metabolic pathways or insulin sensitivity [36, 47]. However, the underlying mechanisms remain unclear and require further investigation. Additionally, chronic heavy drinking may interfere with the accuracy of HbA1c measurements, affecting its association with the new‐onset ADL disability.

Central obesity may be an important pathway by which elevated HbA1c levels contribute to BADL disability [48]. The accumulation of abdominal fat is usually accompanied by chronic low‐grade inflammation and serves as an important source of proinflammatory cytokines (e.g., IL‐6 and TNF‐*α*), which not only induce or exacerbate insulin resistance by interfering with insulin signaling pathways but also promote inflammation—a chronic inflammatory state associated with aging—ultimately leading to impaired tissue function and sarcopenia [49, 50]. This cascade of metabolic dysfunction can significantly increase the risk of BADL disability. Recent studies support this hypothesis. For example, Garrido‐Dzib et al. [51] showed in an elderly Mexican population that central obesity (measured by waist circumference), in combination with elevated HbA1c levels, had a particularly pronounced effect on cognitive impairment and functional health. Similarly, Botoseneanu et al. [13] and Jiang et al. [52] found that elevated HbA1c levels were often associated with increased waist circumference, possibly mediated by chronic inflammation and insulin resistance, which together increase the metabolic burden and accelerate functional decline. The results of the present mediation analysis are consistent with these observations and highlight the critical role of waist circumference in modulating the effects of glucose dysregulation on functional health. These findings highlight the importance of integrating waist circumference management into diabetes care strategies for middle‐aged and older adults. From a public health perspective, targeted interventions focusing on both glycemic control and central obesity may be essential in reducing the incidence of functional disability in aging populations. Future research should further explore the mechanisms underlying this relationship and assess the effectiveness of combined glycemic and weight management strategies in mitigating age‐related functional decline.

This study was the first longitudinal investigation using nationally representative data to examine the association between HbA1c levels and future impairment in performing BADL, demonstrating high generalizability and prospective validity. It also examined the mediating effect of waist circumference in the relationship between HbA1c levels and new‐onset BADL disability, thereby extending the theoretical understanding of how metabolic disorders affect functional health. While this study demonstrated methodological rigor through its sample selection, longitudinal design (with extended follow‐up), and extensive multivariate adjustment to minimize bias, several limitations should be acknowledged. First, despite using data from the China Health and Aging Longitudinal Survey, residual confounding from unmeasured variables not included in the database remains possible. Although this study controlled for multiple confounding factors in the model, the group with low HbA1c levels could consist of individuals whose blood glucose levels had been reduced by insulin therapy or other medical conditions. This could introduce a degree of bias into the findings. Second, although the mediation analysis tentatively identified waist circumference as a mediator between HbA1c levels and BADL disability, the potential underlying mechanisms involving inflammatory markers or other mediating variables were not sufficiently explored. Furthermore, this study did not include fasting blood glucose for comparative analysis, which could limit a comprehensive evaluation of the differences in the roles of various glycemic markers in predicting functional outcomes. Future studies could conduct a comprehensive analysis by combining multiple glycemic markers.

## 5. Conclusion

HbA1c levels showed a significant correlation with new‐onset BADL disability. Elevated HbA1c levels (particularly above 5.2%) predicted an increased risk of developing BADL disability. Central obesity, reflected by waist circumference, may partially mediate this association. These findings highlight that glycemic control with waist circumference management may be an important strategy to reduce the risk of BADL disability.

## Author Contributions

Jing Ren: conceptualization, writing (original draft), and visualization. Tian Lv: formal analysis and data curation. Yong Zhang: writing (review and editing) and visualization. Qinna Mao: conceptualization. All authors have made substantial contributions to the preparation of this manuscript. Jing Ren and Tian Lv contributed equally to this work.

## Funding

This study was funded by the National Social Science Fund of China, No. 25BTY060.

## Disclosure

All authors have read and approved the final version of the manuscript. The manuscript guarantor had full access to all of the data in this study and takes complete responsibility for the integrity of the data and the accuracy of the data analysis.

## Ethics Statement

The study was approved by the Biomedical Ethics Review Committee of Peking University (IRB00001052‐11015), and informed consent was obtained from all participants.

## Conflicts of Interest

The authors declare no conflicts of interest.

## Data Availability

The data that support the findings of this study are available in the China Health and Retirement Longitudinal Study (CHARLS) database at http://charls.pku.edu.cn/.
